# Nosocomial outbreak of multi-resistant *Streptococcus pneumoniae* serotype 15A in a centre for chronic pulmonary diseases

**DOI:** 10.1186/s13756-018-0457-3

**Published:** 2018-12-27

**Authors:** Guido J. H. Bastiaens, Amelieke J. H. Cremers, Jordy P. M. Coolen, Mayke T. Nillesen, Martin J. Boeree, Joost Hopman, Heiman F. L. Wertheim

**Affiliations:** 1 Department of Medical Microbiology & Radboudumc Center for Infectious Diseases, Radboud University Medical Center, Geert Grooteplein Zuid 10, 6525 GA Nijmegen, the Netherlands; 2 Center for Pulmonary Rehabilitation, Radboud University Medical Center Dekkerswald, Nijmeegsebaan 31, 6561 KE, Groesbeek, the Netherlands

**Keywords:** *Streptococcus pneumoniae*, Cross infection, Nosocomial, Antimicrobial resistance, Whole-genome sequencing

## Abstract

We report nosocomial transmission of multi-resistant serotype 15A *Streptococcus pneumoniae* (MRSP) that resulted in two lower respiratory tract infections in a centre for chronic pulmonary diseases. This outbreak highlights the potential for transmission of MRSP among vulnerable patients when laboratory turnaround time is long and patient compliance with transmission-based precautions is low.

## Main text

*Streptococcus pneumoniae* is a major cause of community-acquired pneumonia, bacteraemia and meningitis [1]. Asymptomatic nasopharyngeal colonization is a predisposing factor for pneumococcal infection [2]. Infections arising in hospitalized patients are often regarded as a result of earlier acquisition in the community [2], although hospital outbreaks of susceptible and resistant pneumococci have been reported [3–6]. Here, we describe two risk factors for nosocomial transmission of multi-resistant *Streptococcus pneumoniae* (MRSP) that resulted in two cases of hospital-acquired pneumonia.

**Fig. 1:**
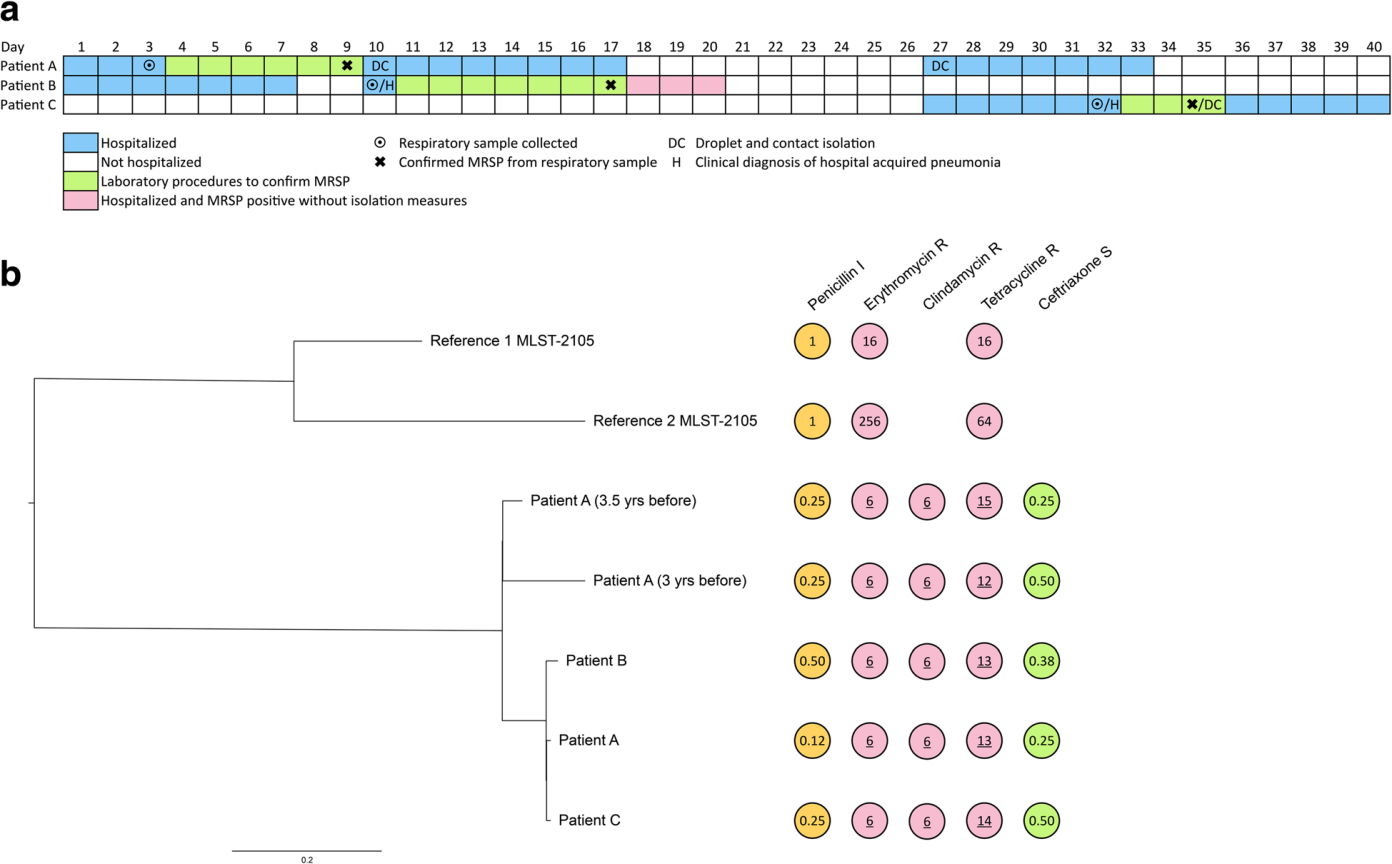
**a**. Timeline of confirmed multi-resistant *Streptococcus pneumoniae* (MRSP) cases. Timeline of patients A, B and C in relation to their admissions, discharges, MRSP positive cultures and infections. **b**. Core SNP-based phylogenetic tree of MLST-2105 serotype 15A strains showing 3 outbreak strains, 2 strains from patient A from 3.5 years ago, and 2 epidemiologically unrelated *S. pneumoniae* strains. Tree is rooted on midpoint. Susceptibility for penicillin and ceftriaxone was determined by E-test and expressed as minimum inhibitory concentration (mg/L). For erythromycin, clindamycin and tetracycline susceptibility was determined by disk diffusion and expressed as zone diameter (mm)

Patient A (index case) was admitted to the Radboud University Medical Center Dekkerswald, a centre for pulmonary rehabilitation, tuberculosis and lung diseases (Groesbeek, The Netherlands) because of psychosocial issues and a mild exacerbation of chronic obstructive pulmonary disease (COPD) probably due to smoking. There were no signs of infection. This patient had been a carrier of MRSP 3.5 years ago, but as MRSP had not been identified in subsequent sputum cultures the patient was regarded as MRSP negative. On day 9 after admission a sputum sample collected on day 3 yielded *S. pneumoniae* that was intermediately susceptible to penicillin and resistant to erythromycin, tetracycline and clindamycin (Fig. 1) indicating that patient A was an asymptomatic carrier of MRSP again. Subsequently, the patient was put on droplet and contact isolation according to protocol and discharged on day 17 (Fig. 1). Patient B was admitted to an adjacent room on the same day as patient A because of an exacerbation of COPD. Patient B was discharged after 6 days; readmitted three days later (on day 10) with a hospital acquired pneumonia, and placed on contact precautions on day 11 because of a rhino- or enterovirus positive throat swab. On day 17 (day 7 after second admission) *S. pneumoniae* with an antibiotic susceptibility pattern similar to that of patient A was isolated from a sputum sample collected on admission. Extension to droplet and contact isolation for MRSP was, however, not installed. Patient B was discharged on day 20. On day 27 patient C was admitted with an exacerbation of COPD and that same day patient A was readmitted with recommended isolation procedures but the patient’s adherence to the instructions for transmission-based precautions was poor. The patients were placed in adjacent rooms. On day 32 patient C was diagnosed with a hospital acquired pneumonia and *S. pneumoniae* with an identical susceptibility profile to that of patient A was isolated from patient C’s sputum, three days later. Following isolation of MRSP, patient C was immediately put in adequate isolation and discharged on day 40.

We suspected nosocomial transmission, since multiple *S. pneumoniae*, isolated from one department, showed identical but atypical colony morphology (non-mucoid, small, greyish colonies) alongside unusual susceptibility profiles (Fig. 1). In the Nijmegen area, between 2000 and 2017 not one invasive pneumococcal disease isolate had displayed concurrent reduced susceptibility to β-lactams, macrolides, lincosamides, and tetracyclines. Our suspicion for relatedness was supported by their consistent serotype 15A that was assessed by Quellung reaction using Pneumococcus Neufeld Antisera (Statens Serum Institut, Copenhagen, Denmark) according to manufacturer’s instructions. To rule out further transmission, we screened patients admitted to the same ward as the MRSP-positive patients and healthcare workers (HCWs) who had contact with them for carriage of MRSP using throat swabs. None of the 14 inpatients, or 78 HCWs screened, carried MRSP, and no further cases of MRSP arose. The importance of hygiene precautions including hand hygiene was re-emphasized. Since 2014 hand hygiene compliance is monitored in Radboudumc Dekkerswald by direct observations according to the 5 moments of the World Health Organization [7]. At the time of the outbreak hand hygiene compliance was 86–93%.

Nosocomial transmission of MRSP was confirmed by whole-genome sequencing (WGS) on day 41 (Fig. 1) showing that each of the outbreak isolates belonged to the rare multi locus sequence type (MLST) 2105 and carried the following antibiotic resistance genes: *tetM* (resistance to tetracyclines) and *ermB* (resistance to macrolides and lincosamides) [8]. Based on core single nucleotide polymorphism (SNP) analysis, all three strains were highly similar and across their > 2 million base pair long genome we detected only 4 SNPs difference at most. Compared to 2 epidemiologically unrelated serotype 15A MLST-2105 pneumococcal isolates, required for outbreak analysis given the large genetic diversity within the *S. pneumoniae* species [9], the 3 outbreak strains clustered together (Fig. 1) and were, on average, 137 SNPs different from the unrelated MLST-2105 strains. This supports the hypothesis that these cases arose from a common source.

The source of infection was presumably patient A. Although community acquisition of this MRSP by patients B and C cannot be fully ruled out, multiple arguments support nosocomial acquisition. The pneumococcal strain concerned is rarely encountered worldwide and is here proven to be communicable by WGS. Furthermore, patients B and C have been directly exposed to air droplets from patient A during regular chats, while no common source or link outside the hospital could be identified (patients were unrelated and living in different areas). Possible transmission via HCWs was investigated, yet no MRSP carriage could be identified among them, and high hand hygiene compliance (directly related to pneumococcal transmission [10]) suggested appropriate attention for hygiene precautions. Transmission to patient B probably occurred while patient A was not immediately isolated due to time needed for bacterial culture and susceptibility testing (Fig. 1). Patient C was probably infected because patient A was not adhering to droplet and contact precautions. WGS supported our suspicion that patient A was the index case, whose MRSP isolated 3.5 years ago showed 8 SNPs difference from the current isolate, compared to 2 SNPs difference between the isolates from the 3 patients involved in this outbreak.

This outbreak highlights two risk factors for nosocomial transmission of MRSP causing hospital-acquired infections (patients B and C). First, time needed for laboratory diagnosis in low-endemic regions may lead to failure to timely install adequate transmission-based precautions. In this case the delay was due to the atypical colony morphology and resistance pattern necessitating confirmatory tests. Secondly, patient compliance with transmission-based precautions may be low despite standard instructions and may require day-to-day audits.

Considering the fact that patient A still turned out to be a MRSP-carrier after 3.5 years and recent evidence suggests that *S. pneumoniae* serotype and drug-resistance are associated with carriage duration [11], additional measures to prevent nosocomial spread in low-endemic regions could include routine screening at admission for patients who were colonized with MRSP previously, and placing them on droplet and contact precautions until carriage of MRSP has been ruled out.

## Abbreviations

COPD: Chronic obstructive pulmonary disease
HCWs: Healthcare workers
MLST: Multi locus sequence type.
MRSP: Multi-resistant *Streptococcus pneumoniae*
SNP: Single nucleotide polymorphism
WGS: Whole-genome sequencing
